# Monthly Summary

**Published:** 1876-09

**Authors:** 


### MONTHLY SUMMARY.

*To Cut Cold Steel with Soft Iron.*—The development of heat by friction has been long known. For some time it has also been known that the operations of rolling and rubbing had the effect of changing the molecular structure of iron and steel. These operations will toughen and compact cold iron, and will harden and condense steel. Some time since Mr. Jacob Reese, of Pittsburg, Penn., had occasion to construct a machine for cutting bars of cold hardened steel. For this purpose he mounted a disc made of soft wrought iron upon a horizontal axis, so as to be rotated with great velocity. With any moderate speed no cutting was produced. But, on giving the disc such a speed of rotation as to cause the periphery of the disc to move with a velocity of about 25,000 feet per minute (nearly five miles) the steel was rapidly cut, especially when the bar to be cut was slowly rotated against the disc. Sparks in a steady stream were thrown off. At first it was supposed that the steel was simply rubbed or ground off. But on examining the pile of accumulated particles beneath the machine they were found to be welded together in the shape of a long cone, similar to the stalagmites in the limestones caves; they were nearly like the spikes of frost as formed in winter on Mount Washington, and illustrated at the Troy Meeting. Real fusion takes place. The steel is melted by the swiftly-moving smooth edge of the soft iron disc, but the disc itself is but little heated. The bar of steel on each side of the cut receives but a slight heat, and the ends are left with a fine smooth blue finish. By this process a rolled, polished, and hardened steel bar of two or three inches diameter, may be cut in two in a few minutes. The soft metal disc of iron used was about forty-two inches in diameter, and three sixteenths of an inch thick. The particles fly off in a thick jet or stream apparently white hot, through which the naked hand may be passed without injury, and a sheet of white writing paper held in the stream for a minute is not burned nor colored in the least. They glance off without burning the hand, having assumed the condition which causes the spheroidal state of liquids.—S. B. Hedrick, *in American Chemist.*

*Carbolic Acid Poisoning.*—Many cases have been recorded of poisoning by carbolic acid, arising both from its internal use and from its external application of it to a raw surface. It gives rise to giddiness, nausea, a feeble pulse, delirium, coma, or collapse, and sometimes to severe spasms, ending in paralysis and death. The post-mortem examination reveals a liquid state of the blood, and a pale and shrunken condition of the lungs. There is intense venous congestion of the brain and its membranes ; and in Neuman's experiments on the lower animals he found that carbolic acid caused congestion of the brain substance, and fatty or granular degeneration of the liver in the lower animals.

Olive oil is stated to be the best antidote, but the combination formed by oils with carbolic acid tends rather to facilitate the absorption of the latter. The administration of albumen which forms with the acid an inert coagulum, is unquestionably a better measure. Husemann considers that saccharate of lime acts more efficaciously, an opinion with which I am inclined to coincide.

Most authorities state that the treatment of a case of poisoning by carbolic acid should consist, first, of the immediate administration of emetics ; but as the alimentary tract is rendered insensible by the local action of the poison, emetics are entirely inoperative and utterly useless. Without doubt, however, the stomach pump should be used without delay, and plenty of milk and the reputed antidotes should be administered.

Carbolic acid is largely used to prevent stenches. When offensive gases are once formed they are not destroyed by carbolic acid, as they are by chlorine or by permanganate of potash ; carbolic acid can only prevent their generation,

The fact of venous congestion of the brain having been observed as a consequence of carbolic acid poisoning has led to the adoption, by Dr. Moslen, of Griefswald, of venesection of the external jugular, with a successfnl result.—*Dr. W. H. Griffith in Med. Press and Circular.*

---

*Fracture of Maxillæ with Profuse Hemorrhage,*—Dr. C. S. Briggs, describes the following case the result of a railroad accident, in the *Nashville Medical and Surgical Journal:*

With Drs. Galbraith and Roscoe, of Goodletsville, who were in attendance, a careful examination of the more seriously injured patient was made, with the following results: William G——, aged 28, tall and muscular, and showing every indication of previous good health. The right side of his face was considerably swollen, and the eye of that side entirely closed by extensive ecchymosis in the cellular tissue surrounding it. A clean cut in the median line, from under the chin, through the lip, laying bare the bone, and the upper and lower front teeth loosened. Suspecting fracture of inferior maxillary bone the finger was introduccd into the mouth, but no irregularity of the dental arches could be distinguished. Careful manipulation failed to detect any fracture of the skull; pupils normal and responsive to light; mental faculties a little impaired; pulse quick, frequent and feeble; temperature lowered a little, and respiration somewhat quickened. Learned that he had bled profusely all the night before from nose and mouth. He could localize no pain, but complained of general aching.

Administered ether, and with the fingers and dressing-forceps, removed the loose teeth. The finger discovered numerous fragments of bone along the upper end lower alveolar processes which were removed with dressing-forceps. Adjusted the edges of the incised wound with several interrupted sutures, and used a wet compress.

Arterial hemorrhage from the alveolar process soon became alarming. in view of the large quantity of blood he had lost the night before, and for some hours all our efforts to check it were vain. Persulphate of iron, ice, and ergot, were not to be had, and we had to resort to cold water, vinegar, continued pressure, cold cloths, pledgets of cotton steeped in a strong alum solution, green hickory-tree leaves (a country remedy,) but without producing any appreciable effect. Finally we used the powdered alum on the end of the finger, frequently renewed, and to our relief the hemorrhage ceased.

Left the patient resting quietly, with directions to apply compress with four tail bandage, and to use morphine should he suffer. Have since learned that the patient has entirely recovered.

The interesting features of this case are the marvellous escape from death, and the comparatively trifling injury received in a fall from such a height, and the obstinate hemorrhage following the wound. This latter is remarkable, especially when we remember that the coagulability of the blood increases in direct proportion to the amount lost, and his friends state that he had lost almost an incredible quantity during the night. Its increased coagulability was well marked, clotting as soon as it fell in the vessel.

---

*Chromic Acid for Warts.*—Three or four applications of this acid will cause the disappearance of warts, however hard, large or dense these may be. The application gives rise to neither pain, suppuration, nor cicatrices, the sole inconvenience being the production of a dark brown color.—*L. Union Medicale.*

*Electro-Magnetism as a Motive Power.*—Professor Henry was the first to point out the practicability of applying electro-magnetism as a motive power, and in illustration of this he constructed an oscillating apparatus, described in the *American Journal of Science* in 1829. The attempts which have been made to turn this power practically to account have been very numerous. Almost or quite the earliest was made by Messrs. Davenport and Cook, of Vermont, in 1836. A machine in model, exhibited by them in New York, attracted much attention, but a working engine, which they subsequently attempted, did not meet their expectations In all these forms of mechanism there is one unavoidable disadvantage which in the infancy of the science was not known, consisting in the fact that the moving magnets generate in each other currents directly opposed to those from which their own magnetic energy is derived; and hence the dynamic power of the engine is not proportional to the static energy of its component magnets. Electro magnetic engines of some power have, in a few instances, been tried and subsequently abandoned not on account of any mechanical failure, but for reasons of economy. One of this description, constructed under the direction of Den Jacobi at the expense of the Emperor of Russia, was employed to propel a boat on the Neva. Another was the electro-magnetic locomotive of our countryman Dr. Charles G. Page. This was remarkable for its original and ingenious method of applying the power, which was by means of solid cylindrical steel magnets rising and descending in the interior of a pile of short helices, the helices being successively thrown in and out of the circuit. With two such engines, Dr. Page drove a car weighing eleven tons and carrying fourteen passengers on a level track at a rate of nineteen miles an hour. Electro magnetic engines can never compete with steam engines in point of economy until it shall be possible to construct batteries in which the materials consumed shall be, weight for weight, a great deal cheaper than coal. Experimentally it has been proved that a grain of coal consumed under the boiler of a Cornish engine lifts 143 pounds one foot high, while a grain of zinc consumed in a battery to move an electro-magnetic engine lifts only eighty pounds to the same height. But it requires the consumption of a number of grains of coal to produce one grain of zinc.—*Harper's Magazine.*

---

*Antiseptic Property of Borax.*—Prof. Dumas presented to the Academie des Sciences a note in the name of Surgeon-Major Bedois, in proof of the antiseptic property of borax. Two pieces of meat were placed, the one in ordinary water and the other in a solution of borax. While the former was shortly in a state of putrefaction, the other remained quite unchanged, the microscope being unable to discover in the solution either wibriones or infusoria of any kind.—*Med. Times and Gazette.*

